# Health Tract, No. 125

**Published:** 1865

**Authors:** 


### HEALTH TRACT, No. 125.

### VENTILATING THEORIES.

A PAPER was read to the French Academy of Sciences in January, 1863, by M. Delbrück, who thinks it "singular that, while all medical men are unanimous in prescribing several cubic meters of pure air for each person sleeping in a room, as absolutely indispensable for health, all animals appear to shun the open air as much as possible, in order to compose themselves to sleep. Thus, the lion and tiger retire to some dark cavern, where the air is confined; the dog goes to his kennel, and thrusts his snout under his belly; birds, to which the open air would appear to be a necessity, whether asleep or awake, retire to some private corner, and put their heads under their wings. Nay, what does the school-boy do, when left in a dormitory aired with particular care. If he finds he can not fall asleep, the first thing he does is to bury his head under the bed-clothes. Hence, if, when awake, we exhale a quantity of carbonic acid, we must inhale a certain quantity of this gas during sleep, just as plants exhale by day the oxygen they absorb during the night."

A writer in *Harper's Magazine* for February, 1863, describing the people of Iceland and their homes, says: "The dark turf walls are pleasantly diversified with bags of oil hung on pegs, scraps of meat, old bottles and jars, and divers rusty-looking instruments for shearing sheep and cleaning their hoofs. The floor consists of the original lava-bed and artificial puddles composed of slops and offal of divers unctuous kinds. Smoke fills all the cavities in the air not already occupied by the foul odors, and the beams, and posts, and rickety old bits of furniture are dyed to the core with the dense and variegated atmosphere around them. This is a fair specimen of the whole establishment, with the exception of the travelers' room. The beds in these cabins are the chief articles of luxury. Feathers being abundant, they are sowed up in prodigious ticks, which are tumbled topsy-turvy into big boxes on legs, that serve for bedsteads, and covered over with piles of all the loose blankets, petticoats, and cast-off rags possible to be gathered up about the premises. Into these comfortable nests the sleepers dive every night, and, whether in summer or winter, cover themselves up under the odorous mountain of rags, and snooze away till morning. During the long winter nights they spend on an average about sixteen hours out of the twenty-four in this agreeable manner. When it is borne in mind that every crevice in the house is carefully stopped up in order to keep out the cold air, and that whole families frequently occupy a single apartment not over ten by twelve, the idea of being able to cut through the atmosphere with a cleaver seems perfectly preposterous. A night's respiration in such a hole is quite sufficient to saturate the whole family with the substance of all the fish and sheep-skins in the vicinity."

The filthiest people in semi-civilized creation are the fishermen of the Ferroe Islands, and yet they live longer, on an average, than any people of the globe, their death-rate being only twelve out of a thousand, of all ages, in one year; in New-York City it has been reported over thirty in a thousand annually. Several years ago, Dr. McFarlane, of New-Orleans, proved by statistics that the filthiest portion of that city, the swamp in the rear, was the last to be attacked with yellow fever, and that it abated there as soon as any where else; he concluded, therefore, that living in water, mud, and filth, where alligators, dogs, cats, mice, and men were in a state of putrefaction, was a preventive of yellow fever, cholera, diarrhea, etc. And yet the common-sense of every man teaches him that pure air and personal cleanliness in tidy habitations must be promotive of health in all ages and in all climes. Much of the error in morals and physics arises from confounding facts and principles with inferences and deductions. A fact is one thing, an inference is another, and often quite distinct. It is a fact that a man who had a chance of stealing a thousand dollars did not do it, but the inference that therefore he is perfectly honest is not legitimate, for, ten to one, the reason he did not do it was because he was not perfectly sure of not being found out. Many a fellow's repentance begins, not with the commission of the sin, but on the instant of his being found to have been a sinner. We must look at whole facts to become truly wise. Yellow fever and other miasmatic diseases cease among the people living in the swamps in the rear of New-Orleans as soon as any where else, simply because hard frosts put an end to it every where; and we know, by having lived on the spot for many years, that it appears in the swamps sooner or later in the season, not according as the people are more or less dirty, but according to the time at which the bottom of the swamp becomes exposed to a hot sun by the previous evaporation of the water which covered it. If there are many heavy rains during the summer or autumn, or a cold summer, or a late subsidence of the Mississippi, or frequent and long "blows" from the lake inland, there will be no epidemic in the "swamp," however severe it may be in the city. The filthy Ferroe Islanders live long, not because their housekeeping is indescribably filthy, but because during the entire summer their homes are abandoned for the fisheries on the sea; and when they return it is so cold that every thing is frozen up, and there is no decomposition of filth and no evaporation of deadly malarias. As to M. Delbrück's new theory of ventilation, or rather no ventilation at all, it is enough to say for the present that man is neither a pig, nor a goose, nor a goat, and that if the breathing of effete carbonic acid gas promoted health, the wise Maker of us all would have given it to us to breathe instead of the pure air of all out-doors. Men may live in spite of bad air, as they sometimes do in spite of being soaked in rum. Besides, there are always antagonizing influences at work, and various modifying circumstances which readily suggest themselves to educated men; meanwhile, let all bear in mind that sleeping in a pure atmosphere, in our latitudes at least, is indispensable to good health and a long life.

